# Incidence and risk factors of axial symptoms after cervical disc arthroplasty: a minimum 5-year follow-up study

**DOI:** 10.1186/s13018-016-0440-8

**Published:** 2016-09-20

**Authors:** Jing Chen, Jia Li, Gang Qiu, Jingchao Wei, Yanfen Qiu, Yonghui An, Yong Shen

**Affiliations:** 1 Department of Spine Surgery, The Third Hospital of Hebei Medical University, Shijiazhuang, 050051 People’s Republic of China; 2 The Key Laboratory of Orthopedic Biomechanics of Hebei Province, The Third Hospital of Hebei Medical University, Shijiazhuang, 050051 People’s Republic of China; 3 Department of Orthopedic Surgery, Hebei General Hospital, 348 Heping Road, Shijiazhuang, 050000 People’s Republic of China

**Keywords:** Cervical disc arthroplasty, Uncovertebral joint ossification, Cervical kyphosis, Range of motion, Axial symptoms, Clinical outcome

## Abstract

**Background:**

The purpose of this study was to investigate whether uncovertebral joint ossification was a risk factor for axial symptoms (AS) after cervical disc arthroplasty (CDA).

**Methods:**

This retrospective study included 52 consecutive patients who underwent CDA for single-level cervical disc disease. To examine possible risk factors for AS after CDA, univariate and multivariate logistic regression analyses were conducted to compare data from the patients with and without AS (the AS and no-AS groups, respectively).

**Results:**

Among the 52 patients examined, AS were observed in 24 patients (46.2 %), including a stiff neck (*n* = 11), neck pain and dullness (*n* = 10), and shoulder pain (*n* = 3). Uncovertebral joint ossification was detected in 22 (42.3 %) patients, including 17 patients in the AS group and 5 patients in the no-AS group. Clinical outcome improved during the follow-up period for the AS group. According to multivariate logistic regression analysis, uncovertebral joint ossification, cervical kyphosis, and range of motion (ROM) at the index level were identified as significant risk factors for AS after CDA.

**Conclusions:**

Satisfactory clinical outcomes were observed following CDA for the treatment of single-level cervical disc disease in the present cohort. In addition, uncovertebral joint ossification, cervical kyphosis, and ROM at the index level were found to affect the incidence of AS after CDA.

## Background

Cervical disc arthroplasty (CDA) is an alternative to anterior cervical discectomy and fusion (ACDF) for the treatment of single-level cervical spine disease. A theoretical advantage of CDA is its capacity to preserve range of motion (ROM), thereby potentially reducing adjacent level stresses [1–3]. However, after CDA, patients after CDA often complain of neck and/or shoulder pain, a stiff neck, or a dull neck ache. These symptoms are, collectively, referred to as axial symptoms (AS). In previous studies, these symptoms have been attributed to lesions of the disc and facet joints or lesions affecting the muscles of the neck and shoulders [4–6]. In a study by Kawakami et al., a relationship between AS and cervical alignment after ACDF was identified, the authors hypothesized that increased height of the anterior vertebral body affected the incidence of AS after ACDF [7].

Heterotopic ossification (HO) is a common postoperative complication of joint arthroplasty and CDA. Many factors affect the clinical results and development of HO, including gender, advanced age, and multi-level CDA. In addition, HO has been found to affect ROM at the index level. However, it has not been found to have a negative influence on clinical outcome [8–10]. More recently, Chung et al. demonstrated that uncovertebral hypertrophy was a significant risk factor for HO after CDA [11].

In addition to these risk factors, we propose that uncovertebral joint ossification may represent another risk factor for AS after CDA. Uncovertebral joint ossification has the potential to alter dynamics at the index level and may also affect clinical outcome, adversely. Therefore, the purpose of this study was to investigate whether uncovertebral joint ossification was a risk factor for AS after CDA.

## Methods

This retrospective study included a total of 52 patients who underwent single-level CDA in the Third Hospital of Hebei Medical University between July 2004 and June 2009. Inclusion criteria were myelopathy and/or radiculopathy from single-level disc herniation in adult patients that was nonresponsive to appropriate nonsurgical treatment for at least 3 months. Patients with previous cervical spine surgery, an active infection, uncovertebral joint ossification, severe spondylosis and/or disc height loss, ossification of the posterior longitudinal ligament, or kyphotic deformity were excluded from this study. This study was approved by the Regional Ethics Committee of The Third Hospital of Hebei Medical University, and all patients signed informed consent forms.

### Surgical technique

For each patient, CDA was performed by the same senior surgeon. Briefly, an anterior approach via a right-side skin incision was used to perform the surgical procedures. The posterior longitudinal ligament was completely excised and the spinal canal and neuroforamen were decompressed. Endplates were prepared according to the Bryan disc milling technique and this created two concave surfaces. The operative site was then routinely irrigated with saline prior to insertion of the devices. Prior to surgery, the appropriate BRYAN cervical disc (Medtronic Sofamor Danek) was selected based on templating and radiographic studies that included computed tomography to assure appropriate placement.

### Evaluation criteria

Clinical evaluations, including radiological and clinical evaluation results, were collected preoperatively and also at each follow-up. When the follow-up period was longer than 5 years, the last set of available data was used for statistical analysis. The modified Japanese Orthopedic Association (JOA) scoring system was used to determine functional status before surgery and at the final follow-up visit. Both the neck disability index (NDI) and visual analog scale (VAS) were used to evaluate neck and arm pain. AS included neck and/or shoulder pain, a stiff neck, or a dull neck ache. Also at the last follow-up, patients were divided into an AS group or a no-AS group according to whether or not they were experiencing AS.

**Fig. 1:**
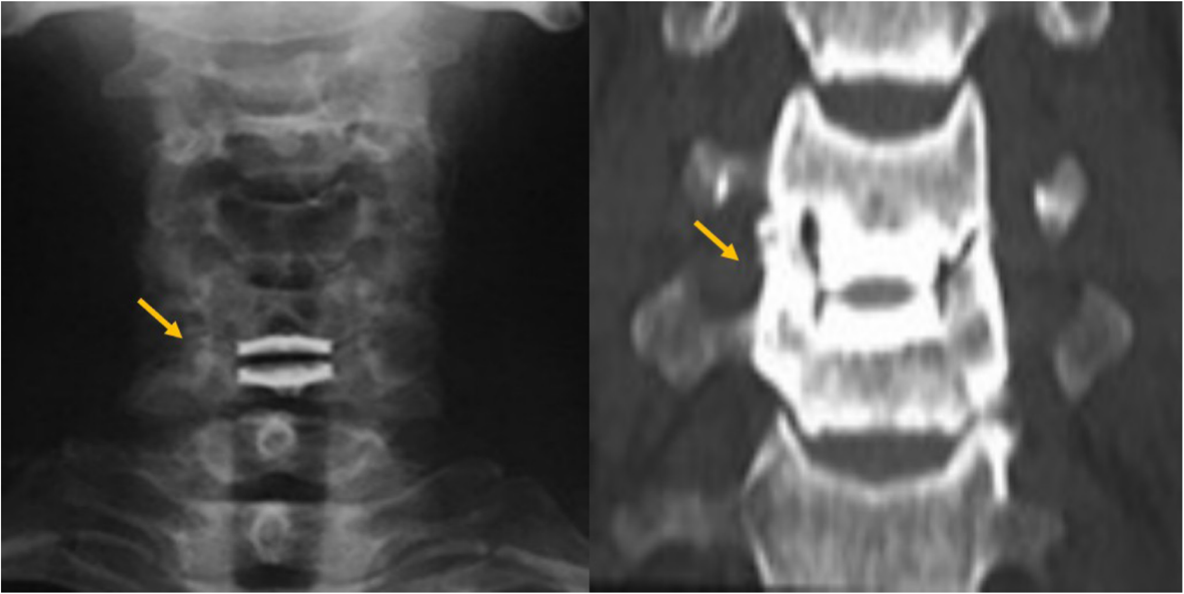
Uncovertebral joint ossification is observed in anteroposterior views from computed tomography and X-ray images

**Fig. 2:**
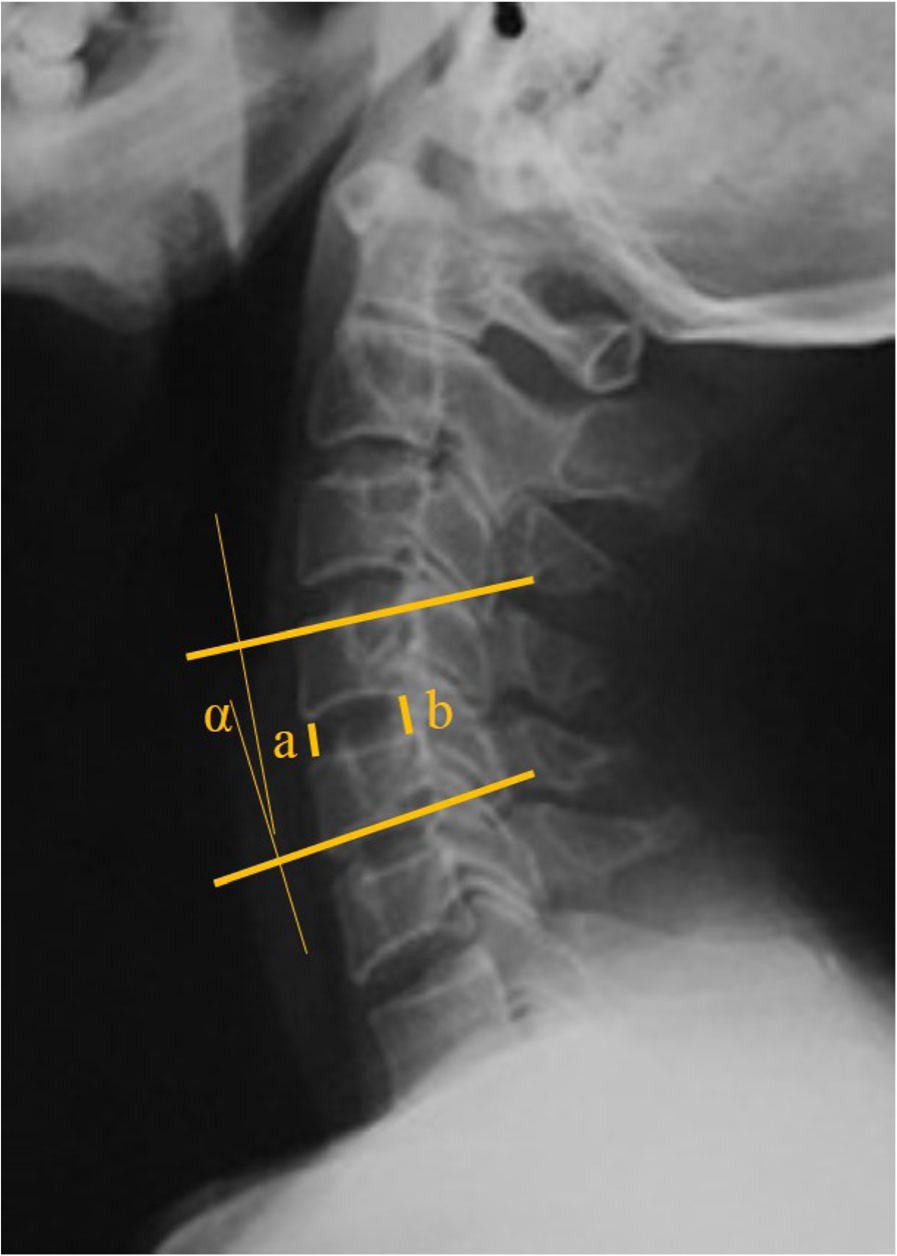
The functional spinal unit angle (*α*) was examined on lateral radiographs and was formed by *lines* drawn at the superior end plate of the cephalad vertebral body and at the inferior end plate of the caudal body. Disc height was defined based on the average values of the anterior disc height (*a*) and the posterior disc height (*b*)

The radiographic evaluation performed included computed tomography and static and dynamic flexion/extension lateral images. The presence of uncovertebral joint ossification was assessed in these images by two independent doctors who were blinded to the clinical outcome of each case (Fig. 1). HO was also assessed from the dynamic flexion/extension lateral images and was graded as described by McAfee et al. [12]. Disc height was defined based on the average heights of the anterior and posterior discs. ROM at the index level was determined by drawing lines between the superior endplate of the adjacent cephalad vertebral body and the inferior endplate of the adjacent caudal vertebral body (Fig. 2). The functional spinal unit (FSU) angle was examined on lateral radiographs and was determined based on the lines drawn at the superior end plate of the cephalad vertebral body and at the inferior end plate of the caudal vertebral body (Fig. 2).

### Statistical analysis

Statistical analyses were performed using SPSS software (version 17.0, Chicago, IL, USA). Significant differences between preoperative and final follow-up measurements were identified by using a paired sample *t* test. An independent *t* test or Chi-square test was used to identify significant differences between groups. Multivariate logistic regression analysis was used to identify risk factors related to the incidence of AS. In all analyses performed, significance was defined as a *P* value less than 0.05. Results are presented as the mean ± standard deviation.

## Results

A total of 52 patients were included in this study and their mean follow-up period was 5.7 ± 0.6 years. AS were observed in 24 patients (46.2 %), and these included a stiff neck (*n* = 11), neck pain and dullness (*n* = 10), and shoulder pain (*n* = 3). None of the patients required additional surgery on either the index level or adjacent levels for recurrent symptoms. HO was observed in 27/52 (51.9 %) patients. Among these patients, 24 patients were classified as grade 1/2, while 3 patients were classified as grade 3. Grade 4, which has the potential to develop into complete arthrodesis, was not observed in the present cohort. Uncovertebral joint ossification was observed in 22/52 (42.3 %) patients of the cohort, with 17 of these patients in the AS group and 5 of these patients in the no-AS group (*P* < 0.05) (Table 1).

**Table 1:** Demographics of the cohort examined

Parameter	AS group	No-AS group	*P* value
Age (years)	46.9 ± 5.3	44.3 ± 6.2	0.571
Gender (male/female)	11/13	12/16	0.829
Operated segment			0.960
C4/C5	5	5	
C5/C6	11	13	
C6/C7	8	10	
Operation time (min)	70.3 ± 9.6	68.5 ± 11.3	0.163
Follow-up (years)	5.9 ± 0.6	5.6 ± 0.3	0.112

In both groups, the JOA, NDI, and VAS scores for neck pain and arm pain were significantly improved in the follow-up period compared to the preoperative scores (*P* < 0.05). Furthermore, except for neck pain (*P* < 0.05), there were no significant differences in these scores between the two groups at the final follow-up (*P* > 0.05). The mean postoperative disc height was 8.2 ± 1.1 mm in the AS group and 7.9 ± 1.2 mm in the no-AS group, and this difference was not significant. In contrast, ROM at the index level for the AS and no-AS groups were 6.3 ± 1.8° and 8.8 ± 2.7°, respectively, and this difference was significant (*P* < 0.05). The FSU angles for the AS and no-AS groups were −0.1 ± 5.2° and 5.0 ± 3.9°, respectively, at the final follow-up, and these differences were also significant (*P* < 0.05) (Table 2).

**Table 2:** Comparisons of outcome factors examined preoperatively versus postoperatively within groups and between the AS and No-AS groups within time points

	AS group		No-AS group	
Outcome	Preoperative	Last follow-up	Preoperative	Last follow-up
JOA	9.8 ± 2.2	14.6 ± 1.6*	10.1 ± 3.6	14.9 ± 2.9*
NDI	46.3 ± 7.2	19.6 ± 3.1*	45.3 ± 5.9	16.1 ± 2.3*
VAS for neck pain	6.9 ± 3.5	3.3 ± 1.2*^,^**	6.7 ± 2.8	1.8 ± 0.9*^,^**
VAS for arm pain	6.3 ± 2.1	1.6 ± 0.7*	6.5 ± 2.1	1.3 ± 0.9*
ROM	7.2 ± 2.1°	6.3 ± 1.8°*	7.9 ± 1.8°	8.8 ± 2.7°*
FSU angle	0.6 ± 0.3°	−0.1 ± 5.2**^,^*	0.7 ± 0.2°	5.0 ± 3.9°**^,^*
Disc height	7.0 ± 1.3	8.2 ± 1.1*	7.1 ± 0.9	7.9 ± 1.2*

*There was a significant difference between baseline and final follow-up; *P* < 0.05

**There was a significant difference between the AS and no-AS groups; *P* < 0.05

°means degree

To compare the relative impact of these variables on the incidence of AS, multiple logistic regression analysis was performed. With a *P* value < 0.1 applied in a univariate analysis, ROM at the index level, uncovertebral joint ossification, and FSU angle were analyzed as dependent variables with a forward stepwise method. Based on this analysis, uncovertebral joint ossification, cervical kyphosis, and ROM at the index level were identified as significant risk factors for AS after CDA (Table 3).

**Table 3:** Multivariate analysis of the risk factors for AS

Risk factor	*P* value	OR	95 % CI
ROM	0.027	1.761	1.1433–2.7144
FSU angle	0.003	1.977	1.3152–2.9719
Uncovertebral joint ossification	0.012	2.437	1.6441–3.6117

*OR* odds ratio, *CI* confidence interval

## Discussion

The results of this study demonstrated that CDA achieved a good clinical outcome, with the overall incidence of uncovertebral joint ossification following CDA being 42.3 %. Furthermore, significant risk factors identified for AS following arthroplasty included uncovertebral joint ossification, cervical kyphosis, and ROM at the index level. CDA is designed to preserve cervical motion and decrease the incidence of adjacent segment degeneration [13]. Occurrence of HO is a common postoperative complication after CDA and has the potential to limit the motion of artificial disc prostheses. When Yi et al. [10] analyzed 170 patients after CDA, with the duration of follow-up being longer than 1 year, the incidence of HO was 40.6 %. Similarly, Lee et al. found that 27.1 % of patients developed HO after a follow-up period of 14 months [14]. More recently, Zhao et al. [15] reported an incidence rate of 69.0 % for HO over a follow-up period of 10 years for a Chinese population. In the present study, 51.9 % of all the patients and operated segments in our series had radiographic evidence of grades 1 or 2 HO at their last follow-up, and 42.3 % of the patients exhibited uncovertebral joint ossification. Van Ooij et al. [16] previously demonstrated that an abnormal movement pattern of segments with a disc prosthesis could explain the incidence of HO. In the present series, postoperative ROM and FSU angles in the AS group significantly differed from the no-AS group (*P* < 0.05). We hypothesize that the cause of HO was abnormal movements in response to segmental cervical kyphosis, and similar outcomes have been observed with lumbar disc arthroplasty [16].

Several studies have reported the influence of gender, age, and multi-level and uncovertebral hypertrophy on HO after CDA [8, 10, 11]. However, it remains unclear whether these factors affect the incidence of AS after CDA. Ebraheim et al. [17] showed that uncovertebral joint osteophytes may cause foraminal stenosis and nerve root compression. In a study by Chung et al. [11], preoperative uncovertebral joint hypertrophy was associated with the occurrence of ROM-affecting HO. Therefore, the authors recommended that uncovertebral joint hypertrophy should be assessed before CDA. Quan et al. [18] observed a trend where patients who developed more extensive HO experienced slightly greater neck pain and had higher arm pain analog scores than patients without HO. However, this difference was not statistically significant. In the present study, ROM at the index level in the AS group was significantly lower compared to the no-AS group (*P* < 0.05). Based on our own experience, a hyperplastic or ruptured posterior longitudinal ligament and anterior and posterior osteophytes of the vertebral body should be removed completely during decompression. In the present study, the endplates were prepared with the Bryan disc milling technique to create two concave surfaces. At the end of the milling process, the resulting outer ridge of bone was able to capture the rim of each shell of the arthroplasty, thereby providing immediate stability. The cortical endplates of the vertebral body also needed to be preserved in order to decrease the risk of subsidence and loosening. Thus, over-milling at the dorsal endplate, the angle of Bryan disc insertion, and loss of lordosis in the disc may contribute to the presence of AS.

After CDA, it is possible that uncovertebral joint ossification accelerates the degeneration of articular surfaces and leads to the growth of osteophytes. This could further hinder ROM, while the development of osteophytes could impinge on nerve roots during movement of the cervical spine. Accordingly, a higher occurrence rate for AS may be related to uncovertebral joint ossification and decreased ROM. In the present study, the AS reported included neck and/or shoulder pain, a stiff neck, or dull neck ache. It remains unclear whether the uncovertebral joint ossification observed will continue to worsen. It is also possible that the biomechanical changes caused by uncovertebral joint ossification could be a contributing factor to AS after CDA.

Previous studies have reported that FSU kyphosis occurs after CDA, and cervical kyphosis was one of the risk factors found to be significantly related to AS after anterior cervical surgery. Correspondingly, Pickett et al. [19] described a patient with postoperative kyphosis that experienced AS and Harrison et al. [20] showed a relationship between cervical kyphosis and AS. In the present study, over-milling of the endplate and asymmetric millings were avoided by selecting an insertion angle parallel to the angle of the native disc space. The structural absence of lordosis in the Bryan prosthesis was a potential cause of segmental cervical kyphosis, and therefore, the posterior longitudinal ligament was removed. Based on our experience, it is hypothesized that there are two main contributing factors to cervical kyphosis, over-milling of the endplate and asymmetric millings. Furthermore, the results of the present study also demonstrated a correlation between segmental cervical kyphosis after CDA and AS.

There were limitations associated with our study. First, the incidence of uncovertebral joint ossification and cervical kyphosis after CDA were reported, although there are many factors after CDA that can affect the incidence of AS. It remains to be investigated whether the impact of uncovertebral joint ossification and cervical kyphosis extends to other artificial disc prostheses. The second limitation of our study was the relatively small patient group size. As a result, identification of the precise incidence and risk factors of AS was limited. Moreover, the degree of uncovertebral joint ossification was not evaluated, and it remains to be determined if it correlates with AS. It is also unclear whether uncovertebral joint ossification worsens with time. None of the present cohort needed additional surgery on either the index level or adjacent levels for recurrent symptoms. In future studies, we will evaluate whether, and to what extent, degradation of the adjacent segment occurs. Biomechanical studies on the occurrence of AS in relation to the degenerative process after arthroplasty would also be essential in identifying the pathogenesis of AS. To establish the exact cause(s) of AS, a larger group of patients treated for CDA, including those undergoing a multi-segment operative technique, are required. In particular, a prospective multi-center study with long-term follow-up would provide very useful information.

## Conclusions

The clinical outcomes of CDA for the surgical treatment of single-level cervical disc disease in this cohort were satisfactory. Furthermore, multivariate logistic regression analysis identified uncovertebral joint ossification, cervical kyphosis, and ROM at the index level to be factors that affect the incidence of AS after CDA.

## Abbreviations

ACDF: Anterior cervical discectomy and fusion
AS: Axial symptoms
CDA: Cervical disc arthroplasty
FSU: Functional spinal unit
HO: Heterotopic ossification
JOA: Japanese Orthopedic Association
NDI: Neck disability index
ROM: Range of motion
VAS: Visual analog scale
